# Callose (β-1,3 glucan) is essential for *Arabidopsis *pollen wall patterning, but not tube growth

**DOI:** 10.1186/1471-2229-5-22

**Published:** 2005-10-07

**Authors:** Shuh-ichi Nishikawa, Gregory M Zinkl, Robert J Swanson, Daisuke Maruyama, Daphne Preuss

**Affiliations:** 1 Howard Hughes Medical Institute, Department. of Molecular Genetics and Cell Biology, University of Chicago, Chicago IL 60637, USA; 2 Department of Chemistry, Graduate School of Science, Nagoya University, Chikusaku, Nagoya 464-8602, Japan

## Abstract

**Background:**

Callose (β-1,3 glucan) separates developing pollen grains, preventing their underlying walls (exine) from fusing. The pollen tubes that transport sperm to female gametes also contain callose, both in their walls as well as in the plugs that segment growing tubes. Mutations in CalS5, one of several *Arabidopsis *β-1,3 glucan synthases, were previously shown to disrupt callose formation around developing microspores, causing aberrations in exine patterning, degeneration of developing microspores, and pollen sterility.

**Results:**

Here, we describe three additional *cals5 *alleles that similarly alter exine patterns, but instead produce fertile pollen. Moreover, one of these alleles (*cals5-3*) resulted in the formation of pollen tubes that lacked callose walls and plugs. In self-pollinated plants, these tubes led to successful fertilization, but they were at a slight disadvantage when competing with wild type.

**Conclusion:**

Contrary to a previous report, these results demonstrate that a structured exine layer is not required for pollen development, viability or fertility. In addition, despite the presence of callose-enriched walls and callose plugs in pollen tubes, the results presented here indicate that callose is not required for pollen tube functions.

## Background

Pollination begins when pollen grains adhere to stigma cells on the female pistil surface. In species with dry stigmas, including *Arabidopsis thaliana*, this interaction is highly selective, allowing binding of only a limited pollen grain set. Pollen adhesion occurs within seconds, is extremely strong, and is mediated by adhesives that reside in the exine, the outer pollen cell wall [1]. Exine fragments washed with organic solvents and salts retain their binding capacity, suggesting that adhesion does not require protein-protein interactions [1]. To discern the genes that mediate *Arabidopsis *pollen capture, we employed simple binding assays, identifying *Arabidopsis *mutants with *l*ess *a*dherent *p*ollen. Here, we cloned *LAP1 *[2], showing it is identical to CalS5 [3,4] a male-specific β-1,3 glucan synthase that plays a role in exine development and pollen tube composition.

Exine patterns are highly variable across plant families, making them an important feature of plant taxonomic classifications and forensic identifications [5]. These lattices are composed primarily of a chemically resistant polymer, sporopollenin, deposited in multiple layers. The ridges and spaces that comprise the exine lattice are established soon after male meiosis when an exine precursor, primexine, is deposited along the plasma membrane of the microspores, just under a layer of β-1,3 glucan (callose) that temporarily separates the developing grains [6,7] Although the molecular mechanisms that yield the vast diversity of exine patterns are poorly understood, species-specific primexine patterning requires coordination between the extracellular callose layer, the plasma membrane, and the underlying cytoskeleton, vesicles and endoplasmic reticulum [5-7].

In addition to *CALS5 *(discussed below), several mutations that affect exine development have been identified, providing opportunities for defining the requirements for sporopollenin synthesis and exine patterning. In the male-sterile *Arabidopsis nef1 *mutant, the pollen surface completely lacks sporopollenin; *NEF1 *encodes a predicted membrane protein that affects lipid accumulation in plastids, potentially in the tapetal cells that surround developing pollen grains [8]. While sporopollenin decorates the surface of *Arabidopsis *male-sterile *dex1 *pollen grains, it is deposited in large disorganized aggregates [7]. *DEX1 *encodes a predicted membrane-associated protein that is required for temporal regulation of primexine deposition [7]. *Arabidopsis qrt *mutants produce tetrads of fused pollen grains, each with a normally patterned cell surface [9,10]. *QRT3 *encodes a endopolygalacturonase required for degradation of the residual mother cell wall surrounding developing microspores [11]. Mutations that alter exine organization have also been identified in other species; for example, a pollen mutant of *Haplopappus gracilis *(yellow daisy) lacks densely packed exine spines, yet is fertile [12]. Genes involved in exine patterning may be particularly diverse, varying in coding sequence or site and timing of expression, and consequently contributing to the evolution of exine patterns [reviewed in [13]].

Callose plays multiple roles in pollen development, not only blanketing microspores as they form independent exine layers, but also as a major constituent of pollen tubes, the polarized extensions that deliver sperm to female gametes. Like most plant cells, pollen tubes contain pectin and cellulose, yet they have an additional layer of callose, a feature that is common to the hundreds of species examined to date [14,15]. Pollen tubes can extend hundreds to thousands of times the pollen grain's diameter, transporting all of the cytoplamsic contents, including the vegetative nucleus and two sperm, to the elongating tip. In order to maintain a manageable cytosolic volume, callose plugs are deposited periodically, separating the growing tip from the evacuated portions [14,15].

Recently plants carrying T-DNA insertions in the *CALS5 *gene, which encodes a male-specific β-1,3 glucan synthase were characterized [3,4]. These studies indicated that *CALS5 *is required to produce the temporary callose walls that separate developing microspores, and that this callose layer is critical for exine formation and fertility [4]. Here, we examined plants carrying three additional *cals5 *alleles; in all cases, we saw a similarly aberrant exine layer, yet the plants retained their fertility. One mutant (*cals5-3*) also lacked detectable callose in its pollen tubes, suggesting it is not essential for their growth or guidance through female tissues.

## Results and discussion

### CalS5 mutants produce adhesion-deficient pollen

**Figure 1:**
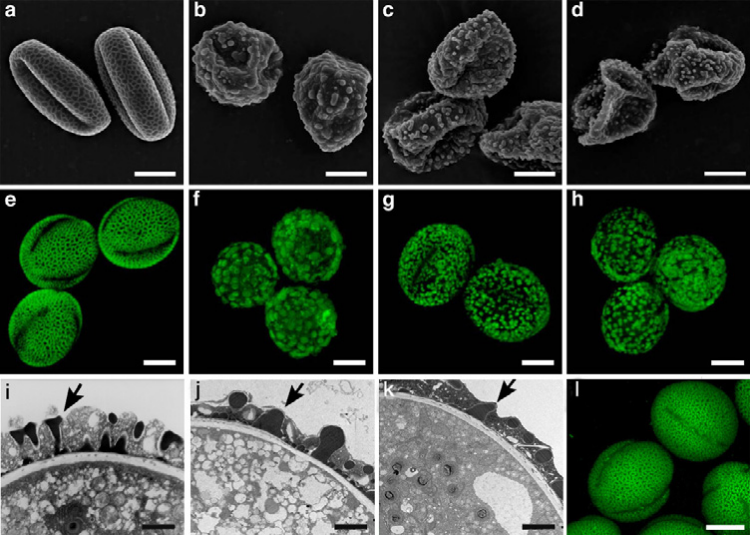
*Arabidopsis *mutants defective in exine pattern formation. Scanning electron micrographs of pollen grains (*a-d*), confocal images of pollen stained with 0.1% auramine O (*e-h,l*), and transmission electron micrographs of mature pollen sections (*i-k*, arrows indicate sporopollenin). Wild type Landsberg (*a*,*e *and *i*), *cals5-3 *(*b*,*f*, and *j*), *cals5-4 *(*c*, *g*, and *k*), *cals5-5 *(*d *and *h*), and *cals5-3 */+ (*l*). Bars, 10 μm (*a-h*, *l*); 1 μm (*i-k*).

We recovered *cals5 *mutations in a screen for genes that mediate *Arabidopsis *pollen capture. Using an assay that measures the number of *Arabidopsis *pollen grains bound to the stigma following a vigorous wash [1], we screened for plants with weak pollen adhesion, surveying a collection of 374 T-DNA insertion lines [2] and ~6700 M2 ethyl nitrosourea (ENU) mutagenized plants. This screen yielded 9 adhesion-defective plants, two of which (*cals5-3 *and *cals5-4*, Ws and Landsberg ecotypes, respectively) had severe pollen surface defects apparent with light, scanning, or transmission electron microscopy (Fig. 1). Consistent with a previously identified role for exine in pollen adhesion [1], exine patterning was altered in the adhesion mutants; the regular network of projections typical of wild type was abolished, resulting in structurally weakened pollen grains that easily collapsed (Fig. 1b,c). Transmission electron micrographs indicated that sporopollenin, the chemically resistant polymer that comprises exine, assembled into irregular aggregates in the *cals5 *mutants (Fig. 1j,k); these structures stained readily with the exine-specific dye, auramine O (Fig. 1f,g). Previous analysis of *cals5-1 *and *cals5-2 *mutants, showed that developing microspores degenerated, with anthers producing only 1–15 pollen grains [4]; in contrast, the mutants we identified generated fertile pollen grains in abundance (see below).

**Figure 2:**
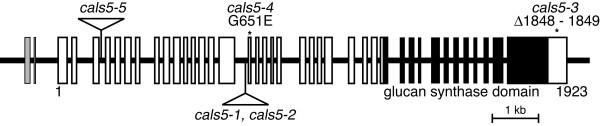
*CALS5 *mutations. The *CALS5 *5769 bp open reading frame encodes a predicted protein measuring 1923 amino acids. Boxes, exons; filled boxes, similar to domains in glycogen synthase; gray boxes, 5' untranslated region. *cals5-3 *is a deletion of amino acids 1848–1849; *cals5-4*, a G651E substitution; *cals5-1*, *cals5-2 *and *cals5-5*, T-DNA insertions.

We mapped *cals5-3 *to chromosome 2; DNA sequencing confirmed mutations in At2g13680 (Fig. 2), the callose synthase that encodes CalS5 [3]. The SIGnAL T-DNA insertion collection [16] contained an additional allele, *cals5-5 *(Columbia ecotype); this insertion also caused an exine defect (Fig. 1d,h). We amplified a cDNA corresponding to *CALS5*, confirming intron-exon boundaries [GenBank:AY337762 and BK001470] and found that the *CALS5 *gene has two additional exons in the 5' UTR region; this annotated structure differs from that previously deposited in GenBank, with 41 exons, instead of the 39 described before [4]. The *cals5-3*, *5-4*, and *5-5 *lesions were localized to exon 41, exon 18, and intron 5, respectively (Fig. 2). In contrast to the null defects identified previously [4], RT-PCR analysis showed that each of mutant lines we characterized produced *cals5 *mRNA, although levels were reduced in *cals5-5*, perhaps reflecting inefficient splicing of intron 5, due to the large T-DNA insertion (not shown). All three *cals5 *exine defects were fully recessive (e.g. Fig. 1l), and pair-wise crosses between each mutant failed to complement.

T-DNA constructs containing a full-length genomic copy of *CALS5 *were lethal in *Agrobacterium tumefaciens*, the organism commonly used for *Arabidopsis *transformation; thus, we were unable to test a transgene for complementation. Nonetheless, because three independent non-complementing mutations were identified, each of which caused a pollen surface phenotype, and two additional exine-deficient alleles have been described [4], it is highly likely that we identified the locus that caused the adhesion defect. The predicted *CALS5 *catalytic domain shares 59 – 69% amino acid identity to 11 other putative *Arabidopsis *callose synthase genes [3,17] and 82% amino acid identity to a callose synthase expressed in *Nicotiana alata *pollen tubes (NaGsl1, [18], [GenBank:AAK49452]).

### *CALS5* expression is male-specific

**Figure 3:**
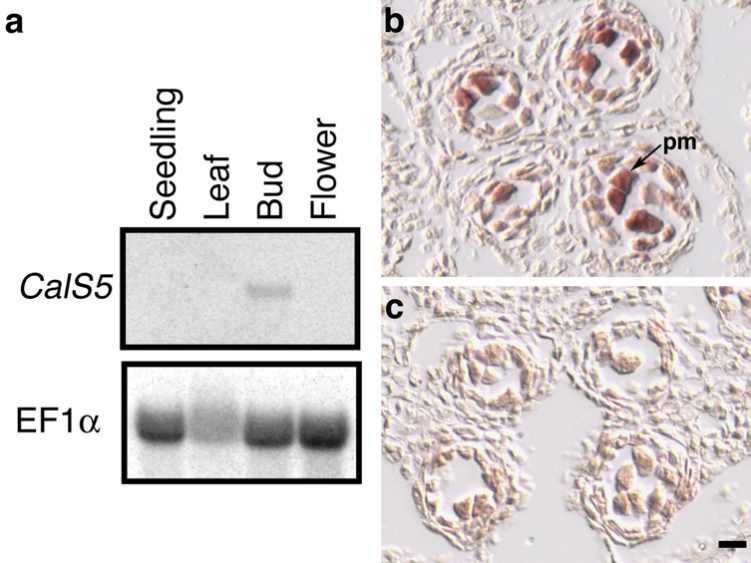
*CALS5 *is expressed in mature pollen and pollen mother cells. Northern blot of mRNA isolated from various tissues and probed with *CALS5 *and EF1α (*a*). *In situ *hybridization of pollen mother cells (pm) within sectioned anthers (*b-c*); *CALS5 *anti- sense (*b*) and sense probe (*c*). Bar, 100 μm.

We detected *CALS5 *expression throughout pollen development, both in pollen mother cells and in developing and mature pollen grains (Fig. 3). By Northern blot analysis, *CALS5 *messages were sufficiently abundant for detection in flower buds, but not vegetative tissues or whole flowers (Fig. 3a), consistent with publicly available microarray datasets showing low, but detectable *CALS5 *expression, beginning in immature flowers and persisting through pollen development [19]. With *in situ *hybridization we showed *CALS5 *mRNA was present at the beginning of pollen development, within pre-meiotic pollen mother cells (Fig. 3b,c). Transgenic plants carrying *CALS5 *fused to reporter genes yielded somewhat different results. Previously, a fusion of 2.2 kb upstream and 20 amino acids of the CaslS5 coding sequence to β-glucuronidase (*GUS*) revealed expression in root tips and in the vegetative vasculature of stems and leaves, and when fused to GFP, showed expression in pollen tetrads, but not pollen mother cells [4]. Here, we fused a 1.8 kb fragment upstream of the *CALS5 *start codon to *GUS*, and found abundant expression in pollen grains starting one day before maturity, but not at earlier stages, and no expression in female reproductive tissues or in other vegetative parts of the plant (not shown); these results were replicated in multiple lines, all of which expressed high levels of GUS in mature pollen. Because direct detection of mRNA showed a different pattern, it may be that an essential promoter element is absent from the fragments used to construct the GUS and GFP fusions. Taken together, the phenotypic and expression data point to a role for *CALS5 *in pollen mother cells and pollen grains, and, although no vegetative phenotypes were observed, expression in vegetative tissues is also possible, due to a putative enhancer more than 1.8 kb upstream of the coding sequence.

### The role of callose in exine formation

**Figure 4:**
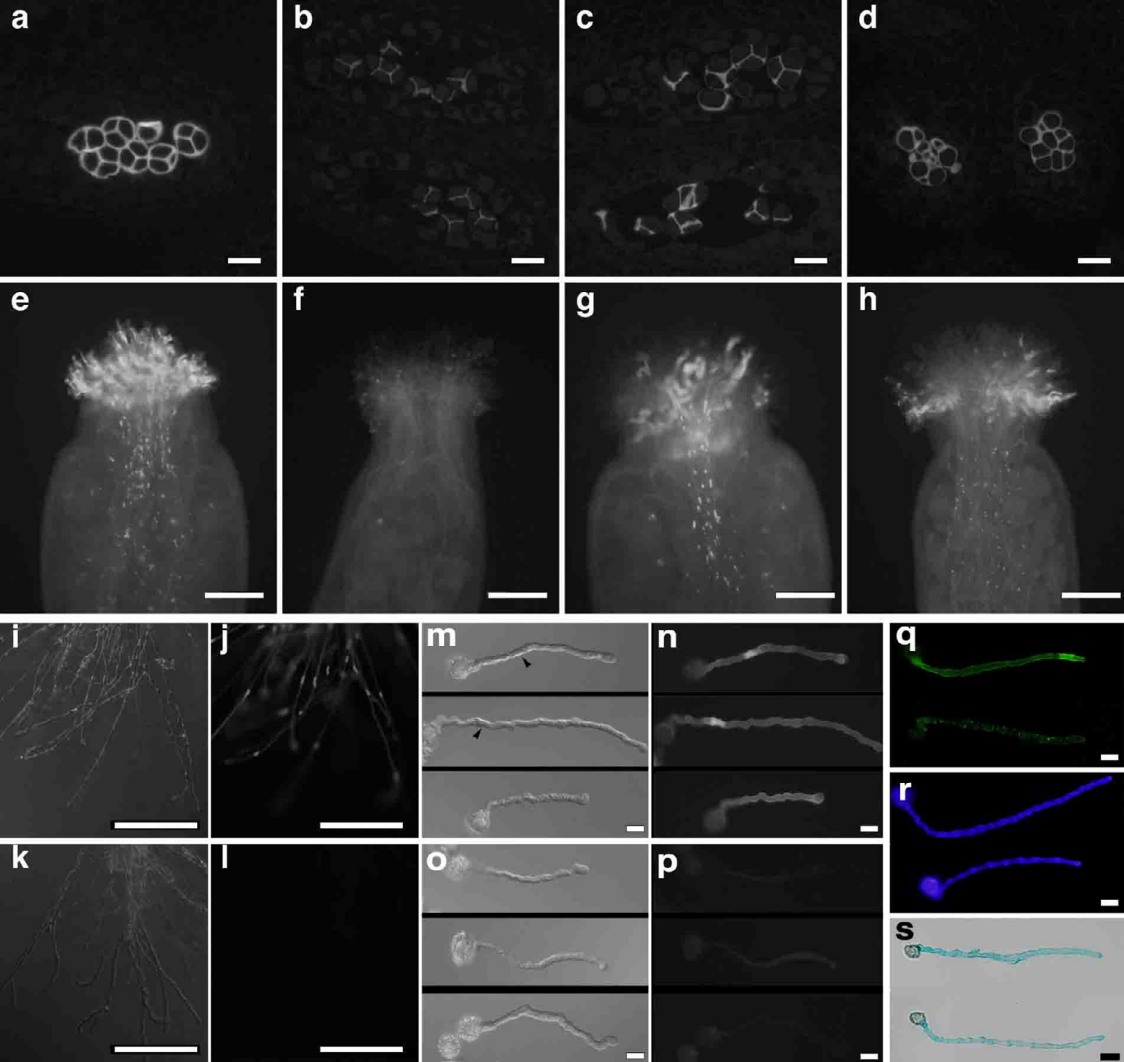
Callose content of *cals5 *pollen tubes. Aniline blue staining (*a-h*,*j*,*l*,*n*,*p*) and differential interference contrast optics (*i*,*k*,*m*,*o*) of sections of tetrad stage anthers (*a-d*), self-pollinated pistils (*e-h*), pollen tubes germinated on an excised stigma and elongating *in vitro *(*i-l*), *in vitro *germinated pollen tubes; arrowheads, callose plugs (*m-p*). Wild-type (*a*,*e*,*i*,*j*,*m*,*n*), *cals5-3 *(*b*,*f*,*k*,*l*,*o*,*p*), *cals5-4 *(*c*,*g*), and *cals5-5 *(*d*,*h*). Wild type (upper) and *cals5-3 *(lower) pollen tubes stained with anti-β-1,3 glucan primary and FITC secondary antibodies (*q*), cellulose staining with calcofluor white (*r*), and pectin staining with Alcian blue 8GX (*s*). Bars, 10 μm (*a-d*, *m-s*); 100 μm (*e-l*).

Tobacco plants overexpressing callase produce inviable pollen with aberrant exine [20,21]. To further explore the relationship between callose formation and exine patterning, we used aniline blue staining to examine the callose that surrounds and separates newly formed *cals5 *microspores (Fig. 4). *cals5-3 *showed a strong defect; unlike wild-type, *cals5-3 *tetrads completely lacked a peripheral callose layer and had little callose between the microspores (Fig. 4a,b). This peripheral callose layer was also missing from *cals5-4 *and *5-5*, but significant callose was present between the microspores (Fig. 4c,d). Interestingly, *qrt2 *mutants have an opposite callose phenotype – they produce fused pollen tetrads with normally patterned exine, and have an intact peripheral callose layer, but discontinuous callose walls between microspores [9]. The *cals5-1 *and *5-2 *alleles appear to be stronger than those reported here; neither peripheral nor interstitial callose walls were detected around microspores, and upon release, developing pollen grains were deformed and often burst [4]. Thus, the *cals5 *allelic series makes it possible to discern varied roles for callose in developing microspores. A complete absence (*cals5-1 *and *5-2*) disrupts both exine formation and pollen viability, while an absence of pheripheral, but not interstitial walls (*cals5-3*, *5-4*, and *5-5*), results in viable pollen with aberrantly patterned exine.

Exine formation requires intracellular functions that nucleate sporopollenin deposition at the pollen plasma membrane; the *Arabidopsis DEX1 *gene product may play such a role [6,7]. Our data indicate that an extracellular callose layer is also required. Conceivably, callose could trap primexine subunits, increasing their local concentration and preventing them from diffusing into the anther locule. These subunits may self-assemble onto a scaffold, nucleated by intracellular components, at the plasma membrane. Subsequently, the removal of callose walls may allow rod-shaped baculae to form on the primexine template. This model is consistent with the *cals5 *phenotype, where disorganized aggregates of sporopollenin collect on the pollen surface (Fig. 1e–k), suggestive of fewer sites of primexine nucleation. It is also possible that callose provides a physical support for primexine assembly, or interacts directly with primexine nucleation sites on the microspore plasma membrane. Distinguishing these models would be facilitated by an *in vitro *system capable of nucleating the assembly of sporopollenin polymers in a manner that recapitulates species-specific patterning.

### The role of callose in pollen tube growth and fertility

Mature pollen contained CalS5-GUS, suggesting a role for callose synthase during pollination. To explore this possibility, we used aniline blue staining to assess callose content in pollinated pistils. Wild type pollen tube walls were readily visualized on the stigma surface and callose plugs appeared as bright punctate dots in the pistil interior (Fig. 4e). These features were also apparent in pollen tubes germinated *in vitro *(Fig. 4i,j[22]); 81.5% of wild-type tubes (n = 351) contained callose plugs, and every tube showed intense callose staining in the outer wall (Fig. 4m,n). In contrast, *cals5-3 *pollen tubes had a severe callose defect; in pollinated pistils, callose was absent from tube walls and bright staining plugs were not detected (Fig. 4f). Consistent with their weaker effect on callose synthesis in pollen mother cells, *cals5-4 *and *cals5-5 *mutants produced pollen tubes with apparently normal callose levels (Fig. 4g,h). We confirmed the *cals5-3 *phenotype *in vitro*; pollen tubes grew normally, yet they lacked aniline staining above background levels and no callose plugs were observed, even with differential interference contrast microscopy (n = 150, Fig. 4k,l,o,p). We also showed that callose-specific staining with an anti-β-1,3 glucan antibody was virtually abolished in *cals5-3 *pollen tubes (Fig. 4q), while cellulose and pectin (Fig. 4r,s) were unaffected. Lastly, to verify that the same genetic defect disrupts callose synthesis in *cals5-3 *pollen mother cells and pollen tubes, we examined 63 F2 plants derived from a cross of *cals5-3 *to wild-type; 15 homozygous mutant lines (verified by PCR) lacked detectable callose in pollen tubes and had aberrant exine, while the remaining *cals5-3 /+ *and *+/+ *lines had normal exine. Together these results indicate that *cals5-3 *is a stronger allele, with severe disruption of callose synthesis both in pollen mother cells and pollen tubes; *cals5-4 *and *cals5-5 *have weaker phenotypes, suggesting a reduction, but not complete loss of function.

**Figure 5:**
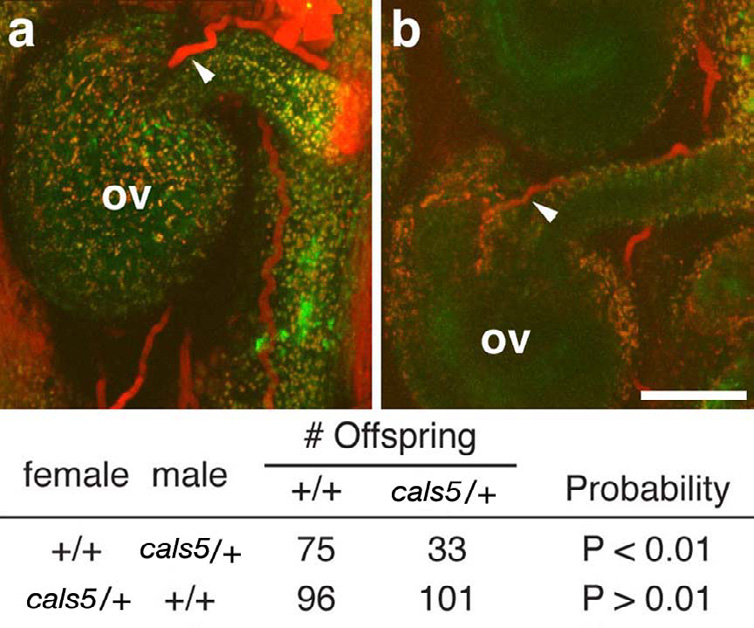
*cals5-3 *pollen tube function. Pollen tubes from self-pollinated wild-type (*a*) and *cals5-3 *(*b*) pistils, stained with 0.1% Congo red and viewed with a confocal microscope. Bar, 50 mm; arrowheads, pollen tubes entering the micropyle; ov, ovule. Offspring ratios determined by PCR from reciprocal crosses of wild-type and heterozygous *cals5-3 *plants (*c*); *P*, probability determined by χ^2^.

The absence of callose in pollen tubes did not eliminate pollen germination, polarized tube growth, pollen tube guidance to the ovules, or the delivery of sperm cells. Despite their deficiency in callose content, *cals5-3 *pollen tubes germinated with normal efficiencies *in vitro *(*cals5-3*, 577/876, 65.9%; wild-type, 466/702, 66.4%), and grew at normal rates (after 6 hrs at 22°C, *cals5-3*, 99 ± 37 μm, n = 110; wild-type, 93 ± 43 μm, n = 140). *In vivo*, *cals5-3 *pollen tubes extended down the length of the pistil at apparently the same rate as wild type, and exhibited wild-type targeting behaviour (n = 300 ovules; Fig. 5a,b). Moreover, self-pollinated *cals5-3 *plants were fertile, producing normal seed sets. This was somewhat unexpected, as callose plugs have been assumed to be essential for limiting the cytoplasmic volume of the pollen tube. Mature *Arabidopsis *pistils measure ~2.5 mm and pollen tubes have 6 μm diameters; thus, we estimate that tubes would undergo at least a 20-fold increase in cytosolic volume if they lacked callose plugs, a factor that would be increased by meandering tube growth. Our results suggest that *Arabidopsis *pollen tubes can tolerate a large expansion in cell volume and that efficient organelle transport can occur within tubes over relatively long distances.

### Callose and pollen tube fitness

*CALS5 *gene expression patterns and *cals5 *mutant phenotypes suggest it functions in both pollen mother cells and pollen tubes (sporophytic and gametophytic tissues [23,24]). The *cals5 *exine patterning defect was fully recessive and therefore showed sporophytic inheritance (Fig. 1l), while *cals5-3 */*+ *plants segregated 1:1 for pollen tube callose content (310:278 callose^+^:callose^-^), indicating gametophytic segregation. This gametophytic role allowed us to test for subtle defects in *cals5-3 *pollen fitness by placing mutant pollen in competition with wild type. First, we noted that self-pollinated *cals5-3 /+ *plants yielded exine-defective progeny at a distorted ratio (1904:362 *CALS5*^+^: *CALS5*^-^; ~5:1). Because flowers from *cals5 *plants consistently produce pollen with exine defects, it is unlikely that this distorted segregation pattern reflects incomplete penetrance; rather, *cals5 *haploid cells are less functional than wild type. This effect was restricted to male gametophytes: crossing *cals5-3 /+ *pistils to wild type pollen yielded normal segregation, while the reciprocal cross showed 31% of the progeny inherited *cals5-3*, differing significantly from the expected 50% ratio (*P <*0.01, Fig. 5c). The ratio of *cals5-3 /+ *: +/+ seeds was not significantly different (*P *> 0.4, χ^2 ^test) in the upper and lower halves of these developing siliques (upper, 37:60; lower, 15:31, 5 pistils sampled), suggesting that *cals5-3 *pollen tube deficiencies were not exacerbated with additional tube growth. Because these crosses did not produce full seed sets, competition was not complete, and it remains possible that the inability of *cals5-3 *pollen to sire as many progeny as wild type is due to slower pollen tube growth *in vivo*. It is also possible that the pollen tube volume increase predicted to result from the absence of callose plugs could account for a loss in pollen competence at these final stages, perhaps diluting key components in an expanded cytosol. Lastly, the heightened demand on systems that transport and secrete materials to the pollen tube tip could reduce the ability of *cals5-3 *pollen tubes to deliver sperm. In either case, the selective pressure of a natural environment could place pollen grains lacking callose at a disadvantage.

## Conclusion

Here we characterized three alleles of the *Arabidopsis CALS5 *gene, dissecting the roles of this callose synthase in pollination. In early pollen development, *CALS5 *has a sporophytic function, forming callose that surrounds pollen mother cells and separates developing microspores. All *cals5 *mutants showed defects at this stage and mutant pollen grains had a pronounced exine defect, providing compelling evidence that callose deposition determines exine patterning. While strong *CALS5 *alleles (*cals5-1 *and *cals5-2*) caused pollen degeneration and sterility [4], we identified *CALS5 *mutants (*cals5-3*, *cals5-4*, *cals5-5*) that are fertile. This work points to the value of an allelic series: other than an exine patterning defect, *cals5-4 *and *cals5-5 *pollen was normal, while *cals5-3 *pollen produced tubes that lacked callose walls and plugs. These differences did not correlate with genetic background; *cals5-1*, *5-2*, and *5-5 *were from the Columbia ecotype, *cals5-3 *was Ws, and *cals5-4 *was Landsberg. Thus, we can define three distinct roles for *CALS5 *in pollen development: i) patterning the exine layer, ii) forming callose in pollen tubes, and iii) preventing pollen degeneration early in development.

The *cals5 *mutants also provide important insight into how variation in callose synthesis could contribute to taxonomic diversity in exine patterns. For example, changes in the site and timing of CalS5 activity could have profound changes on exine structure. In this respect, the callose layer at the pollen mother cell periphery is key; *cals5-4 *and *cals5-5 *retained callose between developing microspores yet produced uniformly aberrant exine. The gametophytic role of *CALS5 *in pollen tubes may provide an added evolutionary constraint on *CALS5 *variation. Direct competition between *cals5-3 *and wild-type pollen showed that callose does provide a fitness advantage at late stages of pollen tube growth. Thus, changes in patterns of callose synthesis at the pollen mother cell must be balanced with the selective pressures that lead to a nearly universal presence of callose plugs; such changes would be possible by differentially modulating sporophytic and gametophytic *CALS5 *expression profiles.

## Methods

### Arabidopsis strains and growth conditions

Although it is caused by a point mutation, *cals5-3 *(Ws-2, CS 2330) was derived from a pool of T-DNA mutagenized seed; *cals5-4 *was isolated from *cer6-2 *(Landsberg *erecta*, CS 6242) mutagenized with 1.25 mM ethyl nitrosourea overnight at 22°C; *cals5-5 *(Columbia, SALK 072226) was obtained from the SIGnAL project [Genbank:BH850992]. Plants were grown on soil with 24 h fluorescent lighting at 22°C, or in a greenhouse. Pollen-stigma adhesion was assayed in self-pollinated M2 pistils as described [1].

### Genetic analysis

*cals5-3 *was not linked to a T-DNA insertion; consequently, we used PCR-based genetic markers  to map *CALS5 *in a population generated by crossing *cals5-3 *to wild-type Columbia. Analysis of 210 F2 plants localized the mutation between nga1145 (10 cM) and mi398 (29 cM) on chromosome 2. The mutation was narrowed to a 122 kb region on BAC F13J11, between T10F5g14B (F-GCTTGTCGGCGATTAAGGTTGGTC; R-GCCCGCATGCAGAAAGAACACC; Hpy188I) and F13J11.8 (F-ACCAATCGGGCCTGACCTTATC; R-CTAGTGGCGTTACACCATTATTTTCAGTTTAG; MspI); restriction digestion cuts only the Columbia products. *cals5-3 *has a 6-bp deletion in At2g13680, which removes an EcoRV restriction site; PCR amplification of the region flanking this site (primers: F-CCGAATGGGAAAACAGCACAG, R-CTTACCACCGGCGAGAATACG) followed by EcoRV digestion was used to score *cals5-3 *alleles in segregating populations.

### RNA analysis

Total RNA was prepared by the TRIzol method [25] or with an RNeasy kit (Qiagen). *CALS5 *cDNA was isolated by RT-PCR from flower bud mRNA and extended by 5'RACE (Invitrogen). PCR products were cloned into pCR2.1 (Invitrogen), and the nucleotide sequence of the *CALS5 *cDNA was determined. Semi-quantitative RT-PCR for 22, 25, 28, and 30 cycles was used to assess the levels of expression of each *cals5 *allele (not shown), using primers that amplified four different portions of the gene (regions adjacent to each *cals5 *mutation, spanning exons 3–5, 6–8, 19–22, and 40–41). For *in situ *hybridization analysis, a 2.8-kb *CALS5 *cDNA SacI fragment was cloned in both orientations into pGEM4z (Promega) and antisense and sense digoxigenin-labeled probes were prepared with *in vitro *transcription using SP6 RNA polymerase. This SacI fragment corresponds to the N-terminal portion of *CALS5 *and lacks significant similarity to other Arabidopsis callose synthases; the probe for Northern blotting included this region, as well as the 5' UTR.

### *CALS5* promoter-GUS fusion

A 0.26-kb HindIII/EcoRV fragment of p35S-2 containing the 35S terminator region was cloned into the HindIII and StuI sites of pGreenII 0229 [26] to give pDM1. The *CALS5 *regulatory region 1815 bp upstream from the translation initiation codon was amplified by PCR using primers 5'-GCGGTCGACACCATATTTTGTCCATGTAAGAC-3' and 5'-GCGAAGCTTCATATGCTCTCCCTGTTACAAAACATTG-3'. The amplified fragment was cloned upstream of the *Escherichia coli GUS *gene. The resultant plasmid, pDM10, was introduced into *Agrobacterium tumefaciens *strain GV3101 with pSOUP [26] by electroporation. *Arabidopsis *was transformed using the floral dip method [27], and transformants were selected on soil spraying the seedlings with 300 μM glufosinate ammonium. Flowers from the T_2 _transgenic plants were stained for GUS activity as described [9]. Samples were cleared in 70% ethanol and photographed.

### Microscopy

Pollen grains were coated with 10 nm of gold (Denton DESK II sputter coater) and viewed with a JEOL JSM-840A scanning electron microscope at 10 kV. LR White resin sections of microspores, transmission electron microscopy, and aniline blue staining were performed as described [20,28]. Pollen germination and tube growth assays were performed as described [29]. Pollen tubes germinated on stigmas or *in vitro *were stained with 0.1% Congo red [22], 1% alcian blue 8GX in 3% acetic acid, or 0.01% calcofluor white (fluorescent brightener 28) in 0.1 M K_2_HPO_4_. For auramine O staining, pollen grains were suspended in 0.1% auramine O and 50 mM Tris-HCl, pH 7.5 and observed under a Zeiss LSM-510 laser-scanning confocal microscope using the filter set suitable for FITC. Indirect immunofluorescence detection of callose was performed on *in vitro *germinated pollen tubes, fixed and treated with cellulase and macerozyme as described [29], labelled with monoclonal mouse anti-β-1,3 glucan antibodies (1:100, Biosupplies, Parkville, Australia), and visualized with Alexa488-labeled goat anti- mouse antibodies (1:1000, Molecular Probes). *In situ *hybridization was carried out as described [30].

## Authors' contributions

SN and GMZ carried out the mutant screens, participated in the mapping and cloning *CALS5 *and helped draft the manuscript. RJS carried out adhesion assays and assembled constructs for complementation. DM performed *in situ *hybridization experiments. DP, SN, and GMZ conceived of this study, participated in its design and coordination and helped to draft the manuscript. All authors read and approved the final manuscript.
